# Fused Filament Fabrication 3D Printing Parameters Affecting the Translucency of Polylactic Acid Parts

**DOI:** 10.3390/polym16202862

**Published:** 2024-10-10

**Authors:** Vladimír Vochozka, Pavel Černý, Karel Šramhauser, František Špalek, Pavel Kříž, Jiří Čech, Tomáš Zoubek, Petr Bartoš, Jan Kresan, Radim Stehlík

**Affiliations:** 1 Department of Applied Physics and Technology, Faculty of Education, University of South Bohemia in Ceske Budejovice, Jeronymova 10, 371 15 Ceske Budejovice, Czech Republic; pcerny@pf.jcu.cz (P.Č.); kriz@pf.jcu.cz (P.K.); cechji01@zf.jcu.cz (J.Č.); bartos@fzt.jcu.cz (P.B.); 2 Department of Technology and Cybernetics, Faculty of Agriculture and Technology, University of South Bohemia in Ceske Budejovice, Studentska 1668, 370 05 Ceske Budejovice, Czech Republic; sramhauser@fzt.jcu.cz (K.Š.); fspalek@fzt.jcu.cz (F.Š.); zoubek@fzt.jcu.cz (T.Z.); jkresan@fzt.jcu.cz (J.K.); stehlr@fzt.jcu.cz (R.S.)

**Keywords:** translucence, FFF, polylactic acid, slicer, layer height, nozzle temperature, fan speed, extrusion multiplier, integrating sphere, luxmeter, ANOVA, HSD test

## Abstract

The effect of 3D printing parameters by Fused Filament Fabrication (FFF) on the translucency of polylactic acid (PLA) parts was investigated. Six different printing parameters were studied: object orientation, layer height, nozzle temperature, fan speed, extrusion multiplier, and printing speed. The commercially available Plasty Mladeč PLA filament and the Original Prusa MK4 3D printer were used for the experiments. The translucency of the printed samples of 50 × 25 × 1 mm dimensions was measured using a luxmeter in an integrating sphere. A total of 32 sample combinations were created. Each sample was printed ten times. The results show that all investigated parameters significantly affect the optical properties of PLA parts. The best measured translucency values were obtained when printing in portrait mode, with a layer height of 0.30 mm, nozzle temperature of 240 °C, fan speed of 100%, 0.75 set extrusion multiplier, and a speed of 40 mm/s. ANOVA was used to statistically evaluate the effect of each parameter on translucency, and statistically significant differences were found between different combinations of parameters (*p* < 0.05). Scanning Electron Microscope (SEM) analysis provided detailed images of the surface structure of the printed samples, allowing for a better discussion of the microscopic properties affecting the translucency. The best print setting has an efficiency of 88% compared to the default setting of 65%. The ability of visible light to pass through the print (translucency) improved by 23%.

## 1. Introduction

Additive manufacturing (AM) plays a pivotal role in Industry 4.0. This cutting-edge technique enables the direct fabrication of prototypes and models for a diverse range of objects, as well as the production of components and acquisition of parts, typically at a cost-efficient rate. These advances accelerate the production process by rapidly transforming 3D designs into functional tangible parts [1,2]. Fused Filament Fabrication (FFF) and Fused Deposition Modelling (FDM) are the most common 3D printing methods [3]. There exists a wide range of materials available for FFF printing techniques, including ABS-based (Acrylonitrile Butadiene Styrene) polymers, polylactic acid (PLA), nylon, high-density polyethylene (HDPE/PEHD), ABS blended with HDPE, polycaprolactone (PCL), polycarbonate (PC), and others [4]. Pure PLA polymer filaments have a notable benefit in their low melting point around 170 °C [5,6] and relatively high specific heat capacity meaning of 1.8 J/g °C at 25 °C compared to values of other polymeric materials which range between 1.2 and 1.8 J/g °C, enabling a slower decrease in temperature while cooling [7,8]. PLA materials also exhibit higher tensile strength, usually between 50 and 70 MPa, and demonstrate a superior elastic modulus, ranging between 2.5 and 3.5 GPa, compared to ABS, Nylon, HDPE/PEHD, PCL, PC, and PET-G, which generally fall within the range of 0.2–3 GPa [9,10]. Additionally, PLA is known for its minimal carbon footprint and low smoke emissions when extruded [11]. PLA is also deemed safe for medical applications due to its nonmetabolic harm [12].

In terms of optical characteristics, PLA products do not possess the inherent translucency of materials such as acrylic or glass; however, Otieno et al. [13] describe the maximum peak of optical conductivity, elucidating the improved insulating and conductive characteristics in contrast to ABS. Another optical characteristic is the refractive index, which ranged from 1.448 [14] to 1.89 at 0.5 THz [15] for PLA-based parts, and the absorption coefficient for this thermoplastic polyester is between 10 and 11 cm^−1^ [15,16], which can be classified under certain terms as both a colourless and transparent material. However, the precise requirements for achieving optimal light transmission in PLA components have not yet been fully discovered. Overall, PLA’s sufficient translucency, degradability, ability to produce complex geometries, and reduced labour requirements offer improved properties for the traditional packaging industry. These advantages in combination also make PLA suitable for potential applications in aerospace [17], construction and civil engineering [18,19], bioengineering [20], optoelectronic industry [21], and robotics [22,23]. The level of translucency and translucency itself in PLA prints can be achieved through the use of formulation and forming (film thickness), postprocessing techniques, additives, and printing parameters [24,25,26]. In particular, this study investigates the effect of 3D printing parameters such as printing (build) orientation, raster angle and layer height, extrusion temperature, printing speed and acceleration, flow rate (extrusion multiplier), and post-processing cooling on the optical properties of the resulting PLA-based products.

**Figure 1:**
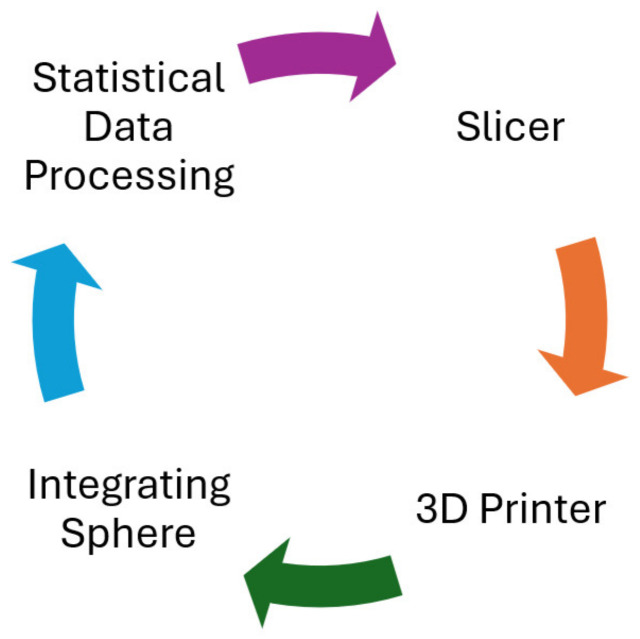
A graphical description of the methodology followed in the work.

An orthogonal design of the experiments (DOE) was carried out. The design of the sample, a rectangular cuboid with defined dimensions, was achieved in the slicer—3D printing software. The slicer was used to define the print, creating the G-code. The G-code was run ten times on the 3D printer. The printed samples were placed in the integrating sphere, and their translucency was repeatedly measured. The data obtained were statistically evaluated. After that, one parameter would be adjusted in the slicer, and the whole cycle would be repeated again. The methodology used in this work is summarised in Figure 1.

A commercially produced filament without any modification was used to create the samples. The filament was used to print samples on the most affordable commercial hobby FFF 3D printer. The most widely used freely available slicer was used to prepare the print. Translucence measurements were performed using a laboratory luxmeter in an integrating sphere.

The Materials and Methods details the materials, equipment, and experimental procedures used in this study. It covers the filament, 3D printer, slicer, integrating sphere, luxmeter, light source, power supply, and printing parameters. Results presents the experimental results, including the effects of different printing parameters on translucency. Statistical analysis is conducted to assess the significance of these effects. The discussion is an integral part of the Results chapter. All values are simultaneously discussed. This section interprets the results and discusses their implications. The findings are compared with the existing literature, and potential mechanisms influencing translucency are explored.

This paper is important because it addresses the effect of FFF 3D printing parameters on the translucency of PLA parts, a topic relevant to both industrial and scientific applications. A novel feature is the detailed investigation of the influence of six specific printing parameters on the optical properties of PLA, which have been insufficiently investigated so far. The results show how printing setup can significantly improve translucency, which can be useful for applications in fields such as optoelectronics, packaging, or bioengineering.

## 2. Materials and Methods

### 2.1. Filament, 3D Printer, Slicer

The filament used for the measurements was Plasty Mladeč (PM) (Hanovice, Czech Republic). The material type was PLA, with a diameter of 1.75 mm ± 0.05 mm in transparent colour in a 1 kg net package (České Budějovice, Czech Republic). The recommended nozzle temperature given by the manufacturer is 200–220 °C. All samples were printed from this package. The material was stored away from infrared (IR), ultraviolet (UV), and visible light in a sealed container with silica gel.

All samples were printed on the Original Prusa MK4 3D printer (Prague, Czech Republic). The printer was purchased as an assembled printer from the manufacturer. The printer is a Cartesian-type, rectilinear XZ-head motion system. The printer is an open-frame type, and it has not been modified in any way. The default filament diameter is 1.75 mm, with a layer height of 0.05–0.30 mm, build volume of 250 × 210 × 220 mm, maximum nozzle temperature of 290 °C, maximum heatbed temperature of 120 °C, and the supported materials are PLA, PETG, ABS, ASA, Flex, HIPS, PA, PVA, PC, PP, CPE, PVB, NGEN, composites, and more.

PrusaSlicer was used to prepare the G-codes. PrusaSlicer version 2.7.1 with up-to-date print profiles was used throughout. No other parameters were changed in the slicer in addition to the discussed parameters; everything was used with the default values.

The sample model was created in PrusaSlicer. The dimensions of all samples are 50 mm wide, 25 mm high, and 1 mm thick.

### 2.2. Integrating Sphere, Luxmeter, Light Source, Power Supply

**Figure 2:**
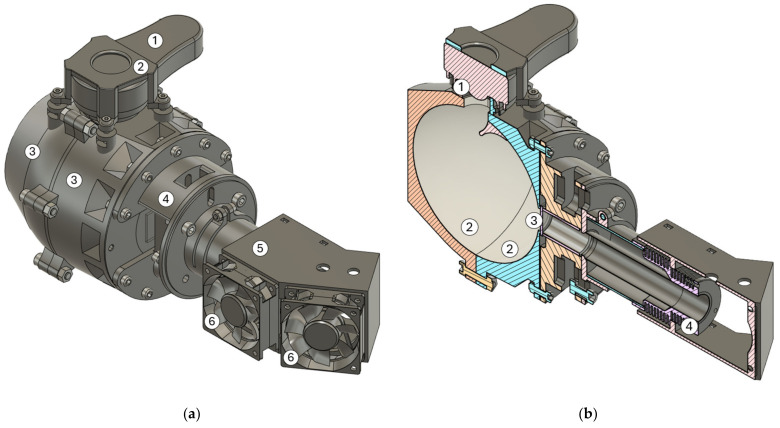
The integrating sphere. The exterior view of the assembly that forms the integrating sphere: (**a**) 1. photometric luxmeter probe; 2. probe holder; 3. half of the sphere; 4. sample holder; 5. fan holder; 6. fan. (**b**) A view of a section of the assembly that forms the integrating sphere: 1. A cosine correction for luxmeter probe; 2. half of the sphere with a barium sulphate coating; 3. the measured sample; 4. the light source heatsink.

An integrating sphere was used to measure the translucency. An integrating sphere is a photometric laboratory measuring instrument that is used to measure the luminous flux of light sources [27]. A custom integrating sphere consisting of two 3D spheres was constructed for measurement (Figure 2a).

The interior of the integrating sphere was coated with a special barium sulphate coating (Figure 2b). Barium sulphate has an ideal reflectivity of 96% to 98% for all types of electromagnetic waves [28]. The integrating sphere was supplemented with a luxmeter and a light source that was powered by a laboratory power supply.

An Extech HD450 (Nashua, NH, USA) luxmeter was used to measure illuminance. The range of illuminance measurement is 0–400,000 lx. The adjustable ranges are 400/4000/40,000/400,000 lx. The illuminance resolutions for these ranges are 0.1/1/10/100 lx. The manufacturer’s stated measurement accuracy is ±5%. The luxmeter can be used as a datalogger, with the ability to record 16,000 values.

A light-emitting diode was used as a source of visible electromagnetic radiation. It was a 36W LED lamp for the H7 socket for 12 and 24 V (Guangzhou, China). The luminous flux is 3800 lm and the colour temperature is 6000 K.

The UNI-T UTP3315TFL (Guangzhou, China) power supply was used to power the light. UTP3315TFL is a linear DC power supply with a classic transformer. The source parameters were as follows: input voltage 230 VAC; adjustable output voltage 0–30 V DC; adjustable output current 0–5 A; output power 150 W; load control mode CV < 0.01% + 5 mV; load control mode CC < 0.1% + 10 mA; accuracy (25 °C ± 5 °C) voltage display < 0.5% + 20 mV; current display accuracy (25 °C ± 5 °C) < 0.5% + 10 mA; CV output control resolution: 10 mV (typical); CC output control resolution: 1 mA (typical).

### 2.3. Printing Parameters in the Slicer

Various print parameters were adjusted in PrusaSlicer. The aim was to achieve the highest translucency. A total of six parameters were examined. The parameters studied in this DOE are shown in Table 1.

#### 2.3.1. Object Orientation

**Figure 3:**
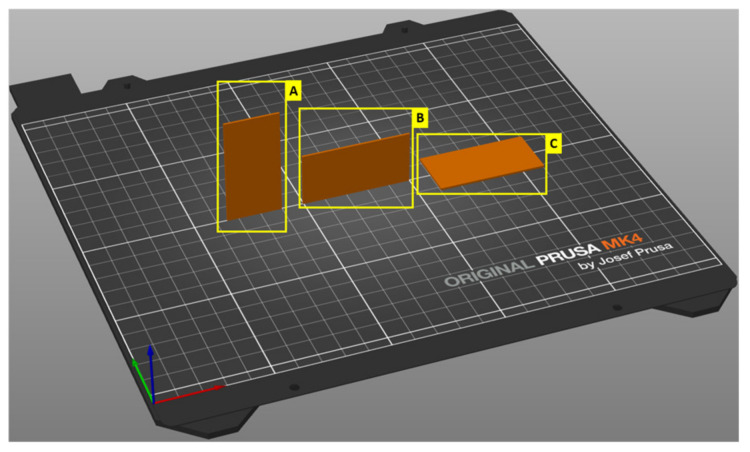
An example of three possible orientations of an object: A (portrait), B (landscape), C (flat).

First, the orientation of the print in relation to the print bed was investigated. Object orientation is crucial for printing. It greatly influences the resulting print quality and controls the print time [29]. Three situations were studied (Figure 3): printing in portrait, landscape, and flat.

#### 2.3.2. Layer Height

**Figure 4:**
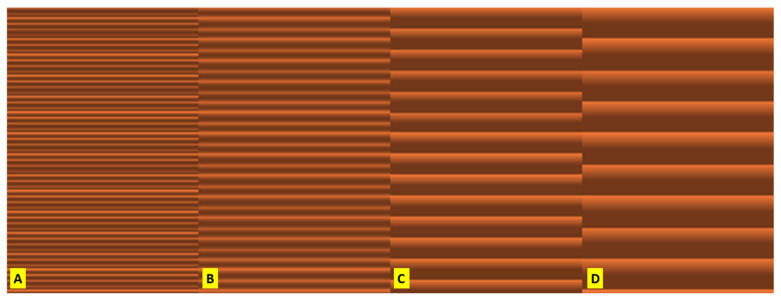
A visualisation of the details of four different layer height settings for a part of the object at the same height in PrusaSlicer: (**A**) 0.05 mm, (**B**) 0.10 mm, (**C**) 0.20 mm, (**D**) 0.30 mm.

The height of the layer is the height of the individual slices/thickness of each layer. Layer height is the main factor that affects the vertical resolution of print time [30]. Four options were studied (Figure 4): 0.05 mm, 0.10 mm, 0.20 mm, and 0.30 mm.

#### 2.3.3. Nozzle Temperature

The temperature of the nozzle determines how well the filament melts, flows, and bonds with the previous layer [31]. Five options were studied: 200 °C, 210 °C, 220 °C, 230 °C, and 240 °C. The default value is 210 °C.

#### 2.3.4. Fan Speed

3D printers come with a dedicated printer fan. By directing ambient air to the extruded filament, the cooling can be dramatically improved. However, with some materials, this could actually make things worse and cause warping or layer separation [32]. It is possible to set the speed as a percentage of the fan’s maximum revolutions per minute (RPM). Three options were studied: 0%, 50%, and 100%. The default value is 100%.

#### 2.3.5. Extrusion Multiplier

The extrusion multiplier allows one to adjust the filament extrusion rate in real time. This allows one to adjust the amount of material that is used to print a particular part of the model [33]. The extrusion multiplier is usually expressed as a decimal number (e.g., 1.0, 0.5, or 2.0). A value of 1.0 means that the filament is extruded at the standard speed. A value less than 1.0 means that the filament is extruded more slowly, reducing the amount of material used for printing. A value greater than 1.0 means that the filament is extruded faster, increasing the amount of material used for printing. Four options were studied: 0.75, 1.00, 1.25, and 1.50. The default value is 1.00.

#### 2.3.6. Speed for Print Moves—External Perimeters

Only speed adjustments for perimeters were discussed. This is because the sample thickness is only 1 mm. Therefore, the object is only made up of perimeters. It is not possible to solve other typical velocities for different types of infills, supports, or bridges.

The speed of the print movement determines how fast the outer wall (shell) of the model is printed. It is usually set slightly slower than the overall print speed to improve the surface quality. A slower speed leads to a smoother and cleaner outer wall surface. On the contrary, too fast a speed can cause unevenness, ripples, and other imperfections [34]. Four options were studied: 20 mm/s, 40 mm/s, 80 mm/s, and 100 mm/s. The default value is 40 mm/s.

Ten samples of each type were printed using the values shown in Table 2.

### 2.4. Statistical Data Processing

Translucency values were measured using a laboratory luxmeter. Analysis of the effect of individual parameters was performed using analysis of variance (ANOVA) at a significance level of 0.05 and Tukey’s honestly significant difference (HSD) test.

### 2.5. SEM Analysis

The fibre structure with different parameters was characterised using a JEOL JSM-7401F field emission scanning electron microscope (SEM) at the Laboratory of Electron Microscopy of the Institute of Parasitology (Czech Academy of Sciences, Ceske Budejovice, Czech Republic).

**Figure 5:**
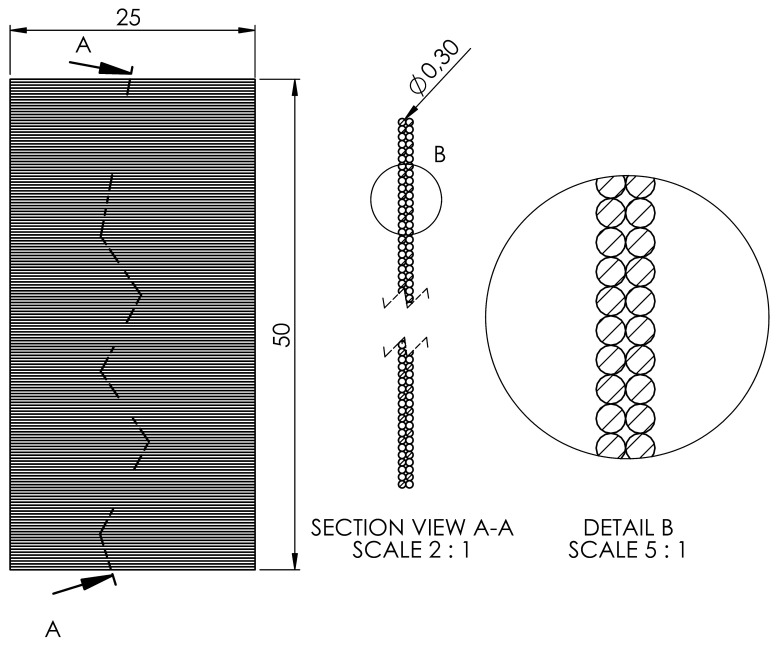
The figure illustrates a random A-A fracture introduced by fracturing after cooling in liquid nitrogen. The section in view of the A-A with the break view shows the internal structure that was analysed by SEM. Detail B is an enlarged detail of a selected section with similar dimensions that was analysed by SEM.

Samples for SEM were prepared by fracturing them in liquid nitrogen. For example, the samples printed on the portrait were fractured crosswise (Figure 5).

## 3. Results and Discussion

The light source was always switched on in advance to stabilise the light flux. The default illuminance value was 300 lx. Each time the sample was inserted, the illuminance was reduced. The illuminance value was always recorded before, during, and after sample insertion. The measured data were always statistically analysed for one selected parameter.

For ANOVA to be valid, three assumptions must be met: the independence of observations, normality of residuals, and homogeneity of variances.

The independence of observations: The data points (translucency measurements) must be independent of each other. Since the study printed multiple samples for each parameter combination and measured their translucency separately, this condition seems to be satisfied.

The normality of residuals: The residuals (differences between observed and predicted values) should follow a normal distribution. However, given the standard deviations provided, this suggests that the data might be approximately normally distributed.

The homogeneity of variances: The variances across different groups should be approximately equal. The variations in standard deviations (especially across parameters like object orientation or layer height) are small, implying that the variances might be similar enough for ANOVA to be valid.

### 3.1. Object Orientation

Studies show that the orientation of an object during printing can affect the mechanical properties and aesthetic appearance of printed objects [35]. For example, printing in portrait orientation exhibits better strength in the layering direction, while printing flat may be more advantageous for achieving a smoother surface finish [36,37,38]. Landscape orientation is often used to optimise printing time and minimise support structures. In addition to surface quality, the printing orientation also affects translucency, as different orientations alter how light interacts with the surface layers. Specifically, portrait orientation tends to allow more consistent light transmission due to more uniform layer stacking, minimising refraction at layer boundaries.

The results of the ANOVA for object orientation show that there are significant differences between the different orientations. As shown in Table 3, the portrait orientation achieves the highest mean illuminance value (246.0 lx) with the lowest standard deviation (0.3 lx), indicating high consistency in the results.

In contrast, the flat orientation has a lower mean illuminance value (194.2 lx) and a higher standard deviation (5.0 lx), which may indicate greater variability in surface quality.

A portrait orientation results in higher mean illuminance because the layers are aligned vertically, reducing scattering and allowing a more uniform light transmission. The lower standard deviation suggests a greater consistency in layer adhesion, minimising variability in translucency. Conversely, a flat orientation introduces more interfaces where light can scatter, leading to lower illuminance and greater variation.

**Figure 6:**
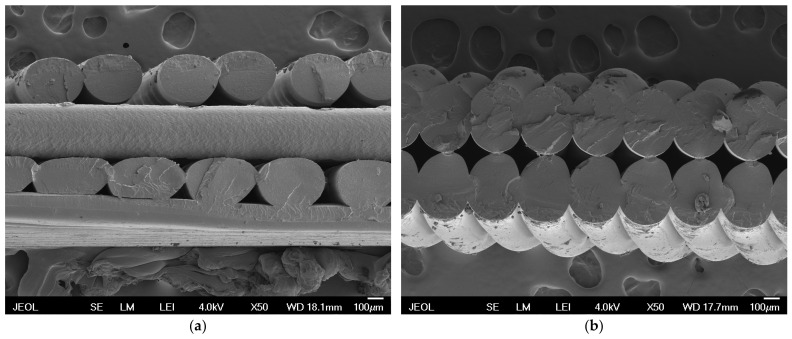
Micrographs obtained by SEM: (**a**) sample 1 (flat OO with one 0.20 mm LH layer and three 0.30 mm LH layers); (**b**) sample 3 (portrait OO with two 0.30 mm LH perimeters).

Micrographs obtained by SEM (Figure 6) provide visual insight into the microstructure of samples printed in different orientations.

Sample 1 (Figure 6a) shows significant differences in the layer distribution compared to sample 3 (Figure 6b). These differences are key to understanding how the different orientations affect the mechanical properties and surface quality of the printed object. Sample 1, printed in a flat orientation with different layers, shows greater irregularities in the microstructure, which may lead to poorer mechanical properties. On the contrary, sample 3, printed in portrait orientation with uniform layers, shows better cohesion and fewer defects between layers. The flat orientation creates twice the number of layers through which light must pass, resulting in multiple refractions of light at the interface of different environments.

### 3.2. Layer Height

Table 4 shows the results of the ANOVA for different layer heights and their effect on the average luminance (lx), a measure of the translucency of the printed object.

The highest translucency was obtained at a layer height of 0.30 mm, followed by 0.05 mm. The lowest translucency was recorded at a layer height of 0.10 mm. These results suggest that to achieve the highest translucency of printed objects, it is advisable to use layer heights of 0.30 mm or 0.05 mm, while using layer heights of 0.10 mm and 0.20 mm leads to a lower translucency. The height of the layer affects the density and arrangement of layers in the printed object. The higher SD for 0.05 mm indicates that at very low layer heights the print is less stable, which can lead to larger differences in translucency. This may be due to the sensitivity of printing to small variations and irregularities when printing fine layers.

With lower layer heights (e.g., 0.05 mm), the print can be more detailed, and the layers are closer together, which can lead to higher translucency if the layers fit together better and create fewer air gaps [39].

Higher layer heights (e.g., 0.30 mm) can also lead to higher translucency if the individual layers contain fewer breaks and transmit light better [40].

The improved translucency at 0.05 mm is due to there being more compact layers with minimal gaps, enhancing light transmission. At 0.30 mm, thicker layers can reduce the number of interfaces, allowing light to pass with fewer refractions. In contrast, 0.10 mm and 0.20 mm create more layers, increasing potential air gaps and light scattering, which reduces translucency.

Different layer heights can affect the properties of the material used, such as shrinkage, expansion, or cohesion between layers [41]. These factors can affect how well the layers transmit light [42].

Different layer heights can cause different material behaviours during cooling and solidification, which affects the resulting translucency [43].

Optical properties such as light scattering and absorption can be affected by the size and arrangement of the layers [44]. For example, smaller layer heights may have more surface areas that scatter light, which may reduce translucency [45]. Higher layer heights may have smoother surfaces, which may improve light transmission and increase translucency [39,44].

### 3.3. Nozzle Temperature

In Section 3.3, the effect of nozzle temperature on the translucency of printed samples is analysed. The results of this analysis are presented in Table 5, which summarises the ANOVA results for different nozzle temperatures (NT).

Nozzle temperature of 240 °C: The average translucency value is 263.5 lx, with a standard deviation of 0.2 lx. This value is the highest among the temperatures tested, indicating that the higher nozzle temperature increases the translucency of the samples. The results show that increasing the nozzle temperature has a positive effect on the translucency of the printed samples.

The causes of differences in translucency values at different nozzle temperatures can be varied and complex. Here are some key factors that can contribute to these results.

Lower nozzle temperatures (e.g., 200 °C): the material may have a higher viscosity, which means it is denser and less fluid [46]. This can lead to poorer material flow during printing, causing uneven layering and reduced translucency.

Higher nozzle temperatures (e.g., 240 °C): the material has a lower viscosity, allowing for better flow and more even layering [47]. This can lead to a better lay-up and higher translucency.

The crystallisation conditions of the polymer may vary depending on the melting temperature [48]. At higher melting temperatures, the melt becomes more homogeneous, which can enhance the optical properties of the printed object. Additionally, slower cooling and solidification at these elevated temperatures allow for improved molecular ordering, leading to increased translucency. Furthermore, higher temperatures promote a better orientation of the polymer chains and reduce crystallinity, which further contributes to the material’s increased translucency [46,47].

Some materials may respond sensitively to temperature changes [49]. Lower temperatures can cause more microscopic defects or air bubbles in the structure of the printed material [48,49]. Microstructural defects, such as bubbles or crystalline inconsistencies, interfere with light transmission by creating multiple interfaces where light scatters or reflects. These defects disrupt the homogeneity of the material, reducing the overall translucency. Bubbles, in particular, cause significant light diffusion, scattering light in various directions rather than allowing it to pass through.

All these factors together can affect the resulting translucency values at different nozzle temperatures. The results obtained suggest that the optimal nozzle temperature to achieve maximum translucency is around 240 °C, which may be due to a combination of improved material flow, reduced defects, and the improved crystalline structure of the material at this temperature.

### 3.4. Fan Speed

Table 6 shows that increasing the fan speed improves the uniformity and cooling quality of the extruded material, resulting in better optical properties (higher average lx value) and less variability in the results (lower standard deviation). The statistical significance of these results is confirmed by a low *p*-value.

The speed of the fan affects the cooling of the extruded material during 3D printing [50]. This cooling affects the quality of the layer and therefore the translucency of the final print [51]. When the fan is turned off, cooling is minimal, which can lead to uneven cooling of the material and, therefore, more light scattering, which reduces the average value (lx) and increases the standard deviation. At a medium fan speed, cooling is more uniform than when the fan is off, resulting in a higher mean value (lx) and a slightly lower standard deviation. At maximum fan speed, cooling is most efficient and uniform, resulting in the highest mean (lx) and lowest standard deviation. Uneven cooling leads to varying levels of shrinkage and layer misalignment at the microscopic level. This causes surface irregularities and introduces areas where light is scattered rather than transmitted. As layers cool unevenly, differences in refractive index between cooled and still-heating regions further disrupt light paths, reducing translucency. Uniform cooling minimises distortion and the layers interconnect better, improving translucency [50,51,52].

### 3.5. Extrusion Multiplier

Table 7 shows the results of an analysis of variance (ANOVA) for the extrusion multiplier (EM) parameter, which adjusts the amount of material extruded by the nozzle during printing.

Higher translucency at lower extrusion multiplier values: a lower extrusion multiplier (0.75) means less extruded material, which can lead to thinner and more uniform layers that transmit light better.

Lower translucency at higher extrusion multiplier values: higher extrusion multipliers (1.25 and 1.50) mean more material is extruded during printing, which can lead to thicker and less uniform layers that scatter and absorb light more, reducing translucency.

A statistical significance *p*-value of 0.000 indicates that the differences between different extrusion multiplier settings are statistically significant, indicating that changes in the extrusion multiplier have a significant effect on the translucency of printed samples.

This analysis shows the importance of optimising the extrusion multiplier to achieve the desired translucency in printed samples, which can be crucial for applications where visual quality and translucency are important.

Less material means thinner layers. Thinner layers can be better connected and more evenly distributed, resulting in higher translucency. With fewer materials, there can be better bonding between layers, which can reduce optical defects, such as gaps or bubbles that reduce translucency [53]. Thin layers cool faster and can better retain their structure, increasing the translucency [54].

Too much material can cause uneven distribution and poor bonding between layers, leading to more defects and reduced translucency [55]. More material leads to thicker layers. These can cause the material to be less homogeneous and more layered, which degrades light transmission and reduces translucency [56]. The thick layers cool more slowly, which can cause the material to shrink or deform more, which degrades optical properties [57].

**Figure 7:**
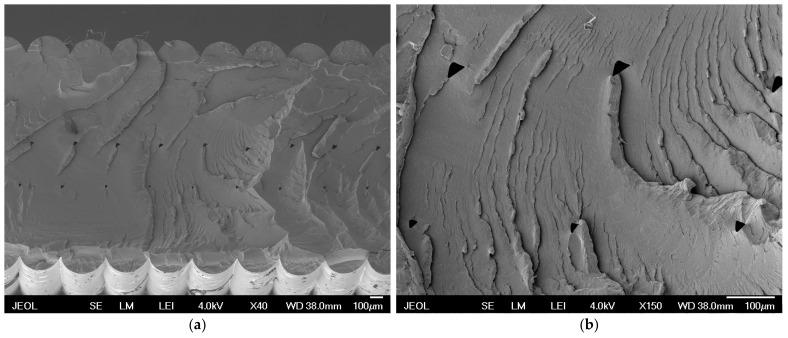
Micrographs obtained by SEM: (**a**) sample 21 (portrait OO, 0.30 LH, 220 NT, 100 FS, 1.50 EM, 40 SOPM); (**b**) detail of space between layers for sample 21.

The images in Figure 7 illustrate the microscopic structures of the printed layers, where the space between the layers can be seen, confirming the above explanations.

At higher extrusion multipliers, the layers are more compact and less permeable to light (Figure 7).

### 3.6. Speed of Print Moves

Table 8 provides the results of the variance analysis for the speed of print moves (SOPM).

A speed of 40 mm/s gives the best results in terms of translucency, with the highest average illumination value (lx). Too high a speed (100 mm/s) leads to lower translucency and higher variability, which may be due to insufficient time for proper curing and smoothing of the layers during printing. To achieve maximum translucency, a print speed of 40 mm/s should be used. A speed of 20 mm/s also gives good results, but slightly lower values than 40 mm/s. Higher speeds above 40 mm/s are not recommended as they lead to significantly poorer translucency results.

At higher print speeds, the material may not have enough time to spread and cure properly, which can lead to uneven layer thickness and consequently reduced translucence [32,40,45]. High speeds can cause insufficient heating of the material, which can affect its ability to bond properly with previous layers [49]. At lower printing speeds, the layers have more time to smooth and cure, resulting in better translucency [53]. In contrast, at higher speeds, microscopic unevenness and defects can occur, reducing translucency [55]. Higher printing speeds can cause a loss of precision in the positioning of the print head, which can lead to inaccuracies in the layering of the material, thereby degrading the optical properties of the printed object [55,57]. Faster printing speeds may mean that the layers do not have sufficient time to cool and cure properly before the next layer is applied, which can lead to the distortion and degradation of the translucency [42,43,49].

A more detailed quantitative analysis of the cooling times at different printing speeds reveals that at slower speeds (e.g., 20–40 mm/s), the cooling is more uniform, promoting better interlayer bonding and reducing defects. Faster speeds (e.g., 80–100 mm/s) result in insufficient cooling time between layers, leading to weaker bonding, increased scattering, and reduced structural stability, which in turn decreases translucency.

### 3.7. Ideal Settings

**Figure 8:**
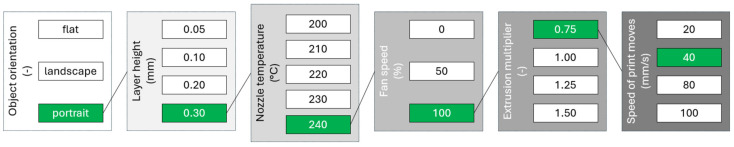
Recommended slicer setting values for the highest translucency.

The recommended values for object orientation, layer height, nozzle temperature, fan speed, extrusion multiplier, and speed of print movements are summarised in Figure 8.

#### 3.7.1. SEM Analysis

**Figure 9:**
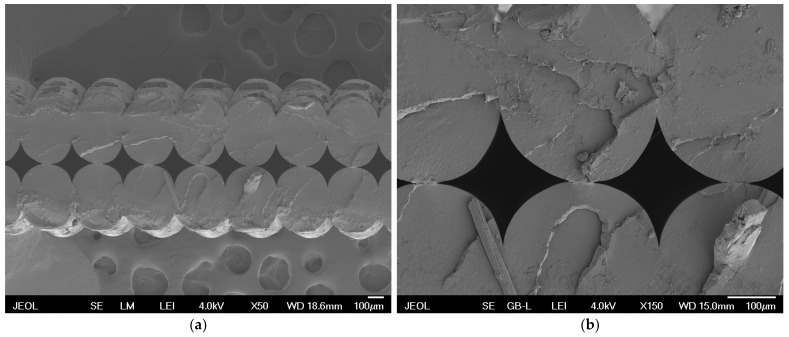
Micrographs obtained by SEM: (**a**) sample 26 printed with the best parameters (portrait OO, 0.30 LH, 240 NT, 100 FS, 0.75 EM, 40 SOPM); (**b**) the detail of the space between the layers for sample 26.

Sample number 26 represents the ideal printing parameters found for the selected printer and the printed material. In measurements, this sample achieved the best translucency values—263 lx out of a possible value of 300. Micrographs obtained by SEM (Figure 9a) show a microscopic view of sample 26. Figure 9a shows that the optimal parameters lead to more uniform and compact layers, which may contribute to higher translucency as a result of better homogeneity and a reduced presence of defects between layers.

Figure 9b is a detailed view of the space between the layers in sample 26. The detailed view shows that, under optimal printing conditions, the layers are well connected with regular shapes of air gaps between the layers. The rounding reaches an almost regular radius. This continuity between layers improves the optical properties of the material, leading to higher translucency.

**Figure 10:**
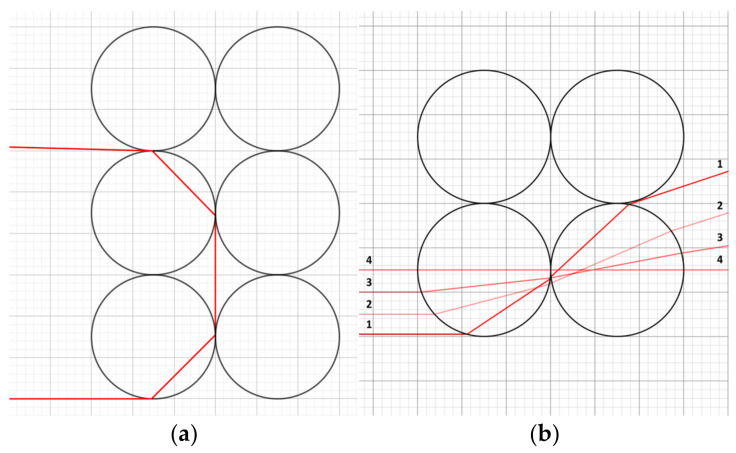
Simulation of refraction of light rays when passing through sample formed by two perimeters created in GeoGebra: (**a**) ray returning back to light source; (**b**) rays passing through to other side of sample.

Let us assume a highly regular and homogeneous structure composed of PLA fibres with an ideal circular cross-section, each having a diameter of 30 micrometres. These fibres are arranged in such a manner that they are in close contact with each other, creating a consistent geometric configuration throughout the sample (Figure 10).

According to [58], the refractive index of PLA at a wavelength of 587.6 nm is 1.459. A light beam incident on an individual fibre at a distance of *h* = 14.72 micrometres from the axis of its circular cross-section undergoes refraction at the interfaces between the fibre and the interstitial voids. Due to the precise alignment and close packing of the fibres in two layers, the beam can be refracted so that it cannot penetrate through the entire thickness of the sample (Figure 10a). This results in the reflection and absorption of light, thereby reducing the intensity of the transmitted light. Conversely, beams directed closer to the centre of the fibre manage to pass through the sample, albeit with scattering (Figure 10b). Hence, approximately 2% of the incident light beams fail to traverse the sample, primarily due to refraction at the fibre interfaces. Additional light intensity attenuation can be attributed to inherent inhomogeneities within the fibre composition, fibre shape variations, and in the fibre junctions’ nature. These factors can introduce slight deviations in the refractive index or incident angles, leading to further scattering and reflection within the sample, resulting in a consequent reduction in light transmission.

#### 3.7.2. Qualitative Comparison Using a Black and White Raster

**Figure 11:**
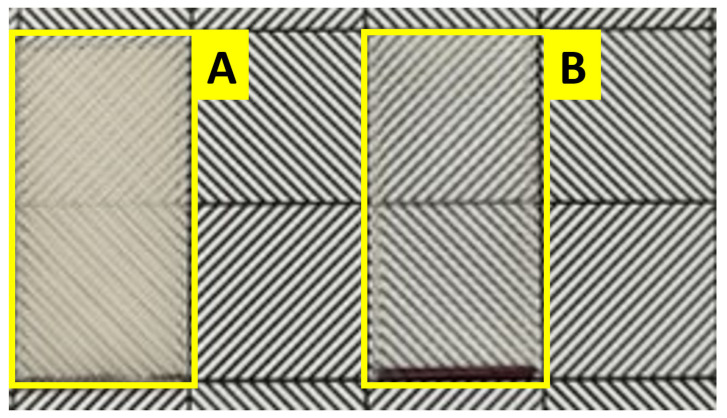
Printed samples placed on paper with a regular geometric pattern. A qualitative evaluation of the different translucency of samples with different printing parameters: (**A**) sample 1, (**B**) sample 26.

For a simple qualitative comparison of the different optical properties of two different prints, a black-and-white raster with regular geometric shapes was used. The lowest and highest quality prints were placed on the raster to illustrate that the qualitative differences are comparable by eye (Figure 11).

#### 3.7.3. Application of Results in Practise—Pythagoras Cup and Tantalus Cup

The name Pythagoras Cup is derived from the name of the ancient Greek philosopher and scholar Pythagoras of Samos [59]. He is said to have invented the cup at the request of the king to prevent people from drinking too much wine. If one was greedy and poured too much wine, the cup would run out [59].

The origin of the name Tantalus Cup is related to Greek mythology [60]. Tantalus was the king of Lydia. Legend has it that he presented his son to the gods to test their omniscience [61]. He was exposed and condemned to an eternity of hunger and thirst. Standing in the water, he could not drink from it, the branches of the trees with their fruits avoiding him. That is why the cup, which opens spontaneously when filled before drinking, bears his name [60,61].

**Figure 12:**
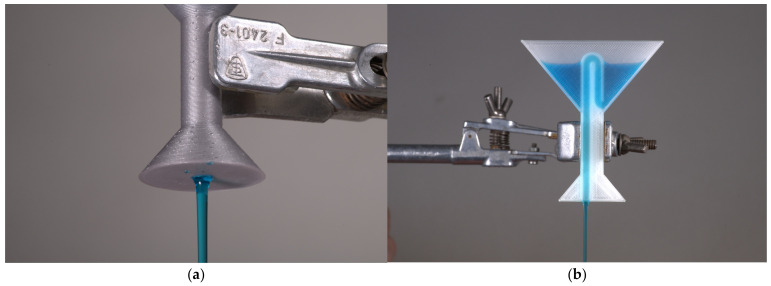
A photo of the Pythagorean Cup model: (**a**) a detailed view of the model made with opaque filament; (**b**) a section view of the model printed with transparent filament with the recommended printing parameters, demonstrating the internal design of the intake system.

For a heuristic demonstration experiment, it is appropriate to 3D print the model from coloured filament with a high infill value (infill = 50%) [62]. The model acts as a black box for which the internal layout should be discussed (Figure 12a) [62].

A further level of understanding is provided by printing from a translucent material, where the internal layout can be partially seen (Figure 12b).

The cup contains two holes, one at the end of the stem and the other inside the compartment where the liquid is poured. The two holes are connected inside the cup to form a suction system. As the liquid is poured in, the suction system, similar to an inverted “U-tube”, is gradually filled. Until the level reaches the highest point in the bend, the suction system is filled only in the upward direction. The moment the liquid level reaches the highest point in the bend, it starts to flow into the descending second part and from there out of the cup [59,62]. Because the other end is below the liquid level in the cup, the pressure differential causes the liquid to drain [59,60,62].

## 4. Conclusions

The study focused on the effect of six printing parameters on the translucency of PLA parts produced by FFF. All the above conclusions apply to the configuration of the equipment already mentioned.

1. The orientation of the object was one of the key parameters that affected the translucency. The results showed that printing in portrait orientation provided better translucency than printing in landscape orientation. Portrait orientation allows for a more uniform distribution of material, which contributes to higher translucency.
2. The height of the layer had a significant effect on the optical properties of the printed parts. An optimum layer height of 0.30 mm was identified as the best compromise between printing time and achieved translucency.
3. The nozzle temperature affects the melting and flow of the material during printing. The best results were achieved at 240 °C (compared to the manufacturer’s recommended temperature of 200–220 °C), which ensured a sufficient melt of the material and minimised internal defects that could reduce translucency.
4. Fan speed plays a role in cooling the material after extrusion. The higher fan speed (100%) resulted in better translucency due to the faster solidification of the material, which minimised deformation and improved surface quality.
5. The extrusion multiplier affects the amount of material extruded. A lower extrusion multiplier of 0.75 resulted in thinner layers of material, which contributed to better translucency by reducing excessive material build-up and internal stresses in the printed parts.
6. Print speed also had a significant effect on the quality and translucency of the printed parts. The optimum speed of 40 mm/s ensured uniform material deposition without defects that could adversely affect translucency. Higher printing speeds led to insufficient heating and the fusion of layers, which impaired translucency.
7. The combination of these parameters resulted in optimum conditions for printing translucent parts. The best translucency values were obtained with a portrait orientation, a layer height of 0.30 mm, a nozzle temperature of 240 °C, a fan speed of 100%, an extrusion multiplier of 0.75 and a print speed of 40 mm/s. This optimised setting resulted in a translucency of up to 88% compared to the default setting’s 65%. The ability of visible light to pass through the print (translucency) has improved by 23%.

## 5. Application of the Results in Practise

Our findings have practical relevance for industrial applications where a high translucency of printed parts is required. The results have been used by the teaching aid community, where optimised print parameters have improved the visibility of translucent parts of models, for example, for fluid mechanics.

For further research, we recommend investigating the effect of other printing parameters and their combinations on the translucency of printed parts. Further research should also focus on other materials used in 3D printing and their specific parameters, which may affect the optical properties of the resulting parts.

## Figures and Tables

**Table 1:** Parameters and values used in DOE.

Printing Parameter	Option 1	Option 2	Option 3	Option 4	Option 5
Object orientation (OO)	portrait	landscape	flat		
Layer height (LH)	0.05 mm	0.10 mm	0.20 mm	0.30 mm	
Nozzle temperature (NT)	200 °C	210 °C ^1^	220 °C	230 °C	240 °C
Fan speed (FS)	0%	50%	100%^1^		
Extrusion multiplier (EM)	0.75	1.00 ^1^	1.25	1.50	
Speed of print moves (SOPM)	20 mm/s	40 mm/s ^1^	80 mm/s	100 mm/s	

^1^ Default value.

**Table 2:** The design of the experiment: object orientation (OO), layer height (LH), nozzle temperature (NT), fan speed (FS), extrusion multiplier (EM), and the speed of print moves (SOPM).

No.	OO (-)	LH (mm)	NT (°C)	FS (%)	EM (-)	SOPM (mm/s)	No.	OO (-)	LH (mm)	NT (°C)	FS (%)	EM (-)	SOPM (mm/s)
1	flat	0.30	210	100	1.00	40	17	portrait	0.30	220	0	1.00	40
2	landscape	0.30	210	100	1.00	40	18	portrait	0.30	220	50	1.00	40
3	portrait	0.30	210	100	1.00	40	19	portrait	0.30	220	100	0.75	40
4	flat	0.20	210	100	1.00	40	20	portrait	0.30	220	100	1.25	40
5	landscape	0.20	210	100	1.00	40	21	portrait	0.30	220	100	1.50	40
6	portrait	0.20	210	100	1.00	40	22	portrait	0.30	220	100	1.00	20
7	flat	0.10	210	100	1.00	40	23	portrait	0.30	220	100	1.00	80
8	landscape	0.10	210	100	1.00	40	24	portrait	0.30	220	100	1.00	100
9	portrait	0.10	210	100	1.00	40	25	portrait	0.30	240	100	0.75	20
10	flat	0.05	210	100	1.00	40	26	portrait	0.30	240	100	0.75	40
11	landscape	0.05	210	100	1.00	40	27	portrait	0.30	240	100	0.75	80
12	portrait	0.05	210	100	1.00	40	28	portrait	0.30	240	100	0.75	100
13	portrait	0.30	200	100	1.00	40	29	portrait	0.30	240	100	1.50	20
14	portrait	0.30	220	100	1.00	40	30	portrait	0.30	240	100	1.50	40
15	portrait	0.30	230	100	1.00	40	31	portrait	0.30	240	100	1.50	80
16	portrait	0.30	240	100	1.00	40	32	portrait	0.30	240	100	1.50	100

**Table 3:** The results of the illuminance value of the ANOVA for object orientation (OO).

Object Orientation	Average (lx)	Standard Deviation	*p*-Value
flat	194.2	5.0	0.000
landscape	240.8	3.2	
portrait	246.0	0.3	

**Table 4:** The results of the ANOVA for layer height (LH).

Layer Height (mm)	Average (lx)	Standard Deviation	*p*-Value
0.05	245.4A	1.3	0.000
0.10	229.4B	0.4	
0.20	232.4B	0.7	
0.30	246.0A	0.3	

Values with the same letter are not significantly different from each other.

**Table 5:** Results of ANOVA for nozzle temperature (NT).

Nozzle Temperature (°C)	Average (lx)	Standard Deviation	*p*-Value
200	242.1	0.5	0.000
210	246.0	0.3	
220	248.7	0.2	
230	247.8	0.2	
240	263.5	0.2	

**Table 6:** Results of ANOVA for fan speed (FS).

Fan Speed (%)	Average (lx)	Standard Deviation	*p*-Value
0	243.9	2.0	0.000
50	246.9A	1.8	
100	248.7A	0.2	

Values with the same letter are not significantly different from each other.

**Table 7:** Results of ANOVA for extrusion multiplier (EM).

Extrusion Multiplier (-)	Average (lx)	Standard Deviation	*p*-Value
0.75	255.6	0.5	0.000
1.00	248.7	0.2	
1.25	228.4	3.8	
1.50	222.6	2.5	

**Table 8:** Results of ANOVA for speed of print moves (SOPM).

Speed of Print Moves (mm/s)	Average (lx)	Standard Deviation	*p*-Value
20	251.8	0.5	0.000
40	255.6	0.5	
80	231.9	2.3	
100	223.9	5.1	

## Data Availability

The original contributions presented in this study are included in the article. Further inquiries can be directed to the corresponding author.
